# Chest pain unit implementation in a multidisciplinary hospital: a retrospective observational study

**DOI:** 10.3389/fcvm.2026.1762236

**Published:** 2026-07-08

**Authors:** Ayagyoz Umbetzhanova, Gulmira Derbissalina, Dias Vakpayev, Zhanagul Bekbergenova, Vitaliy Koikov

**Affiliations:** Department of Family Medicine with Course of Evidence-Based Medicine, Astana Medical University, Astana, Kazakhstan

**Keywords:** acute coronary syndrome, chest pain, emergency service, hospital, myocardial infarction, outcome assessment (Health care)

## Abstract

**Background:**

Acute chest pain is a common reason for emergency department visits and requires rapid differentiation between life-threatening and non-life-threatening conditions. Chest Pain Units (CPUs) were developed to improve diagnostic efficiency and optimize management of patients with suspected acute coronary syndrome (ACS). This study evaluated clinical and healthcare utilization patterns before and after CPU implementation in a multidisciplinary hospital in Kazakhstan.

**Methods:**

A retrospective case–control study was conducted at a 250-bed tertiary hospital. Two consecutive periods were analyzed: pre-implementation (2015–2017) and post-implementation (2018–2020) of CPU. Patients presenting before CPU implementation received standard emergency department care (non-CPU group, *n* = 1,595), whereas those presenting after implementation were managed in the CPU (*n* = 2,121). All consecutive patients with suspected ACS (ICD-10 I20–I22) were included (total *n* = 3,716). Final diagnoses were based on discharge ICD-10 coding. The CPU protocol included standardized assessment, electrocardiography, serial cardiac troponin measurements, and HEART pathway risk stratification. Outcomes included invasive procedures, mortality, hospital length of stay, and direct treatment costs.

**Results:**

Baseline characteristics showed a higher proportion of men in both groups (*p* = 0.015), with no significant age difference (*p* = 0.066). The CPU group had a higher PCI rate (OR 1.43, 95% CI 1.26–1.64, *p* < 0.001), whereas diagnostic angiography was more frequent in the non-CPU group (OR 0.76, 95% CI 0.67–0.87, *p* = 0.02). No significant difference was observed for CABG (*p* = 0.07). AMI diagnoses were more frequent in the CPU group (37.6% vs. 25.1%; OR 1.80, 95% CI 1.57–2.09, *p* < 0.001), while unstable angina was more common in the non-CPU group (OR 0.50, 95% CI 0.48–0.64, *p* < 0.001). Mortality did not differ significantly between groups (2.4% vs. 2.2%, *p* = 0.543). Direct treatment costs were significantly higher following CPU implementation (*p* < 0.001).

**Conclusion:**

CPU implementation was associated with higher rates of AMI identification and increased PCI use compared with the traditional emergency department approach. CPU-based pathways may improve diagnostic accuracy and management patterns in patients with suspected ACS.

## Introduction

Cardiovascular diseases (CVD) remain the leading cause of mortality worldwide, accounting for 17.8 million deaths annually and representing a major global healthcare burden (1). Acute chest pain is one of the most common reasons for emergency department (ED) visits and requires rapid differentiation between life-threatening and non-life-threatening conditions. Acute coronary syndrome (ACS) accounts for approximately 5%–20% of chest pain presentations in emergency care (2). Delayed or inaccurate diagnosis may lead to adverse clinical outcomes, inappropriate discharge, increased healthcare costs, and medico-legal consequences (3–5).

To address these challenges, Chest Pain Units (CPUs) were developed as specialized, protocol-driven short-stay units designed to optimize the evaluation and management of patients presenting with acute chest pain. Typically integrated within or adjacent to EDs, CPUs use standardized diagnostic pathways including serial electrocardiography, cardiac biomarker testing, and validated risk stratification tools to rapidly identify or exclude ACS (6, 7). Their primary objectives are to reduce diagnostic delays, improve patient outcomes, minimize unnecessary hospital admissions, and optimize healthcare resource utilization (6). Since their introduction in the 1980s, CPUs have been increasingly adopted worldwide, particularly in Europe and the United States, and are supported by national and international clinical guidelines due to their demonstrated effectiveness in improving emergency cardiac care pathways (8–10). Evidence suggests that CPU implementation may reduce mortality, shorten hospital stay, improve diagnostic accuracy, and lower overall treatment costs (11–13).

Risk stratification tools such as the HEART pathway have further strengthened CPU-based management by enabling early identification of patients at low and high risk for major adverse cardiac events. Their use has been associated with safer early discharge strategies and improved operational efficiency (14–17).

Despite growing international evidence supporting the use of Chest Pain Units, data from Central Asia and other developing healthcare systems remain limited. This study aimed to evaluate clinical and healthcare utilization patterns before and after CPU implementation in a single multidisciplinary hospital setting in Kazakhstan, including diagnostic characteristics, revascularization rates, in-hospital mortality, hospital length of stay, and direct treatment costs.

## Methods

A retrospective case–control study was conducted at a 250-bed multidisciplinary hospital (single-center study). The study included two consecutive time periods: a pre-implementation period and a post-implementation period of the CPU, allowing comparison of patient management and outcomes before and after its introduction. Patients in the pre-implementation period received standard care in the traditional emergency department (non-CPU group) and were considered the control group. Following the CPU implementation in 2018, all patients presenting with chest pain were directly admitted to the CPU for initial assessment and constituted the case group (CPU group).

All consecutive patients discharged with a diagnosis corresponding to ICD-10 codes I20–I22 during the study period were included. No formal sample size calculation was performed. Patients with incomplete medical records were excluded from analyses where relevant variables were missing. The study retrospectively extracted data from electronic medical records of patients admitted through the emergency department with a primary referral diagnosis of acute coronary syndrome (ACS). Data collection was performed after completion of patient care. A predefined set of variables was obtained, including demographic characteristics, clinical presentation, diagnostic findings, procedural interventions, in-hospital outcomes, and direct treatment costs. The investigation covered two distinct periods: from 01/01/2015 to 12/31/2017 (1,595 patients) for the control group (utilizing non-CPU approach), and from 01/01/2018 to 08/01/2020 (2,121 patients) for the case group (facilitated through CPU assistance with special protocol). The protocol included standardized assessment of patients presenting with chest pain, incorporating clinical evaluation, electrocardiography, serial cardiac troponin measurements, and risk stratification using the HEART score. Based on these findings, patients were categorized into risk groups and managed accordingly. The cumulative sample size for the study comprised 3,716 individuals. Final classification of unstable angina and acute myocardial infarction was based on discharge ICD-10 codes.

The comparative analysis encompassed several key parameters, including the number of diagnostic angiogram, PCI (stenting or balloon angioplasty), coronary artery bypass grafting (CABG), duration of hospital stay, direct treatment costs, mortality rate and the proportion of confirmed diagnoses during angiography.

### Statistic used

Statistical analysis was performed using StatTech v. 4.1.0. Quantitative variables were assessed for normality using the Kolmogorov–Smirnov test. Quantitative variables following non normal distribution were described using median (Me) and lower and upper quartiles (Q1 – Q3). Comparison of frequencies in the analysis of multifield contingency tables was performed using Pearson's chi-square test (for expected values greater than 10). Mann–Whitney U-test was used to compare two groups on a quantitative variable whose distribution differed from the normal distribution. Differences were considered statistically significant at *p* < 0.05. To account for potential confounding between the study groups, a propensity score was estimated using binary logistic regression. The model included age, sex, diagnosis, and procedural interventions as covariates. The estimated propensity score was used to adjust for baseline differences between pre- and post-Chest Pain Unit groups Multivariable logistic regression analysis was subsequently performed to evaluate the association between Chest Pain Unit implementation and in-hospital outcomes. Adjusted odds ratios (OR) with 95% confidence intervals (CI) were reported.

### Ethical approval

Local ethical committee approval was received.

## Results

Data on demographic characteristics and main study variables are shown below in Table As shown in Table 1, there were found statistically significant differences in gender (*p* value 0.015) and no significant difference on age distributions between two groups (*p* value-0.066). Men were more frequently hospitalized with a diagnosis of acute coronary syndrome.

**Table 1:** Baseline characteristics.

Characteristic	Non-CPU (control group)	CPU (case group)	OR (95% CI)	*P* value
Number of patients, total	1,595	2,121	NA	NA
Male	1,019 (63.9%)	1,436 (67.7%)	0.84	0.015*
Female	576 (36.1%)	685 (32.3%)		
Age, Me [IQR]	60.00 [54.00; 68.00]	61.00 [54.00; 69.00]		0.066*
Diagnostic angiogram	605 (37.9%)	674 (31,7%)	0.76 [0.665–0.874]	0.02
PCI with stenting/BA*, cases	583 (36.5%)	960 (45.2%)	1.43 [1.257–1.640]	<0.0001
CABG*, cases	407 (25.5%)	487 (22.9%)	0.87 [0.748–1.012]	0.0713
Unstable angina, cases	1,196 (74,9)	1,324 (62.4%)	0.5 [0.48–0.64]	< 0.0001
AMI*, cases	397 (24.8%)	795 (37.6%)	1.8 [1.567–2.089]	< 0.0001
Mortality rate	38 (2.4%)	46 (2.2%)		0,543
Hospital length of stay, days, Me [IQR]	7.00 [6.00; 10.00]	7.00 [5.00; 10.00]		< 0.001*
Direct cost, average per case in local money (tg*), Me [IQR]	216,206.26 [216,206.26; 893,312.59]	893,312.59 [240,918.07; 995,415.89]		< 0.001*

BA-balloon angioplasty, PCI-percutaneous intervention, CABG-coronary artery bypass graft, AMI-acute myocardial infarction, tg- tenge; *- *applied method: Pearson's chi-square test.*

### Procedures

**Figure 1:**
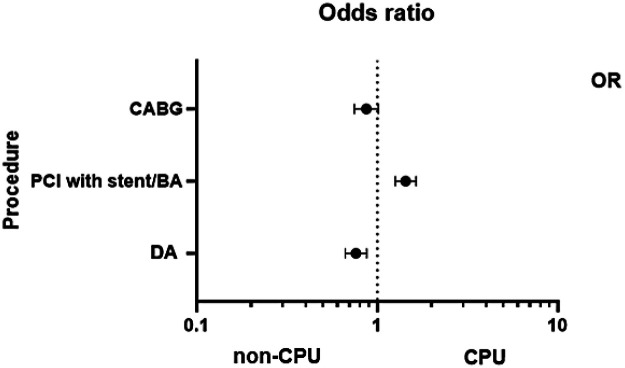
Odds ratio calculated for each procedure in case (CPU) and control (non-CPU) groups (made with graphPad prism 10.2.0).

Then data were analyzed using Mann–Whitney U-test for compare means in case and control groups according to the interventions and procedures performed (Table 1). The analysis showed that the proportion of diagnostic angiogram was higher in the control group, indicating a greater number of procedures performed without subsequent revascularization of the culprit artery and a larger cohort of patients who could be managed conservatively [OR = 0.76, 95% CI (0.665-0.874), *p* value 0.02]. There were no significant differences between CABG cases in both group [OR = 0.87, 95% CI (0.748-1.012), *p* value 0.07]. PCI with subsequent stenting/BA of coronary artery was performed significantly frequent in case group [OR = 1.43, 95% CI (1.257-1.640), *p* < 0.0001], indicating a higher proportion of patients undergoing PCI in the CPU group. Figure 1 displays Odds ratios indicating the likelihood of each procedure being performed in the CPU group compared to the control group.

### Final diagnosis accuracy

Final discharge diagnoses were analyzed in patients with acute coronary syndrome (ICD-10 I20–I22). In the non-CPU group, unstable angina was diagnosed in 1,196 patients (74.9%), while acute myocardial infarction (AMI) was diagnosed in 397 patients (25.1%). In the CPU group, unstable angina was diagnosed in 1,324 patients (62.4%), and AMI in 795 patients (37.6%).

**Figure 2:**
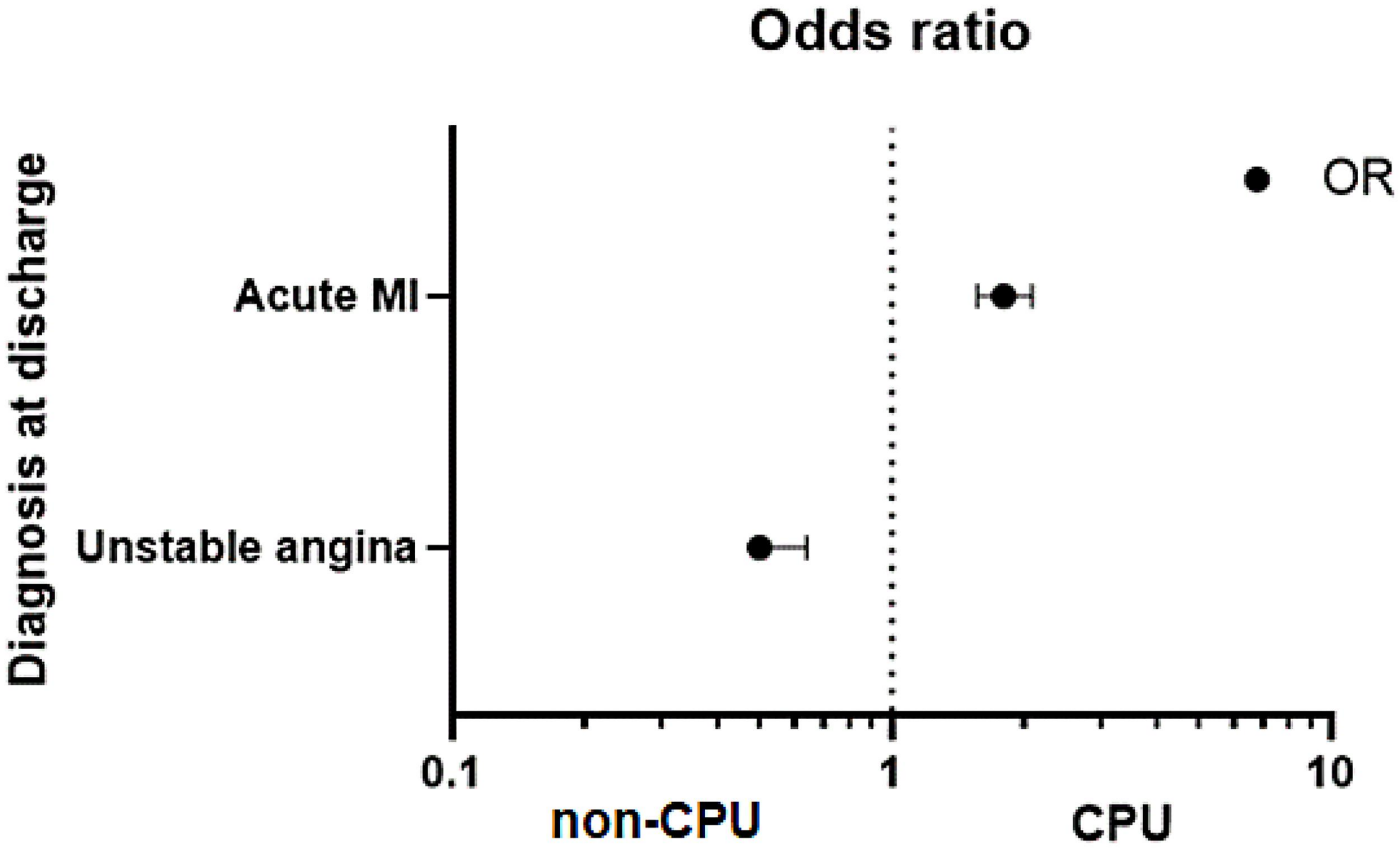
Odds ratio calculated for final diagnosis in case (CPU) and control (non-CPU) groups (made with graphPad prism 10.2.0).

The distribution of final diagnoses differed between groups. The odds of unstable angina were lower in the CPU group (OR 0.50, 95% CI 0.48–0.64, *p* < 0.001), whereas the odds of AMI were higher in the CPU group (OR 1.80, 95% CI 1.57–2.09, *p* < 0.001) (Figure 2).

### Hospital stay

**Figure 3:**
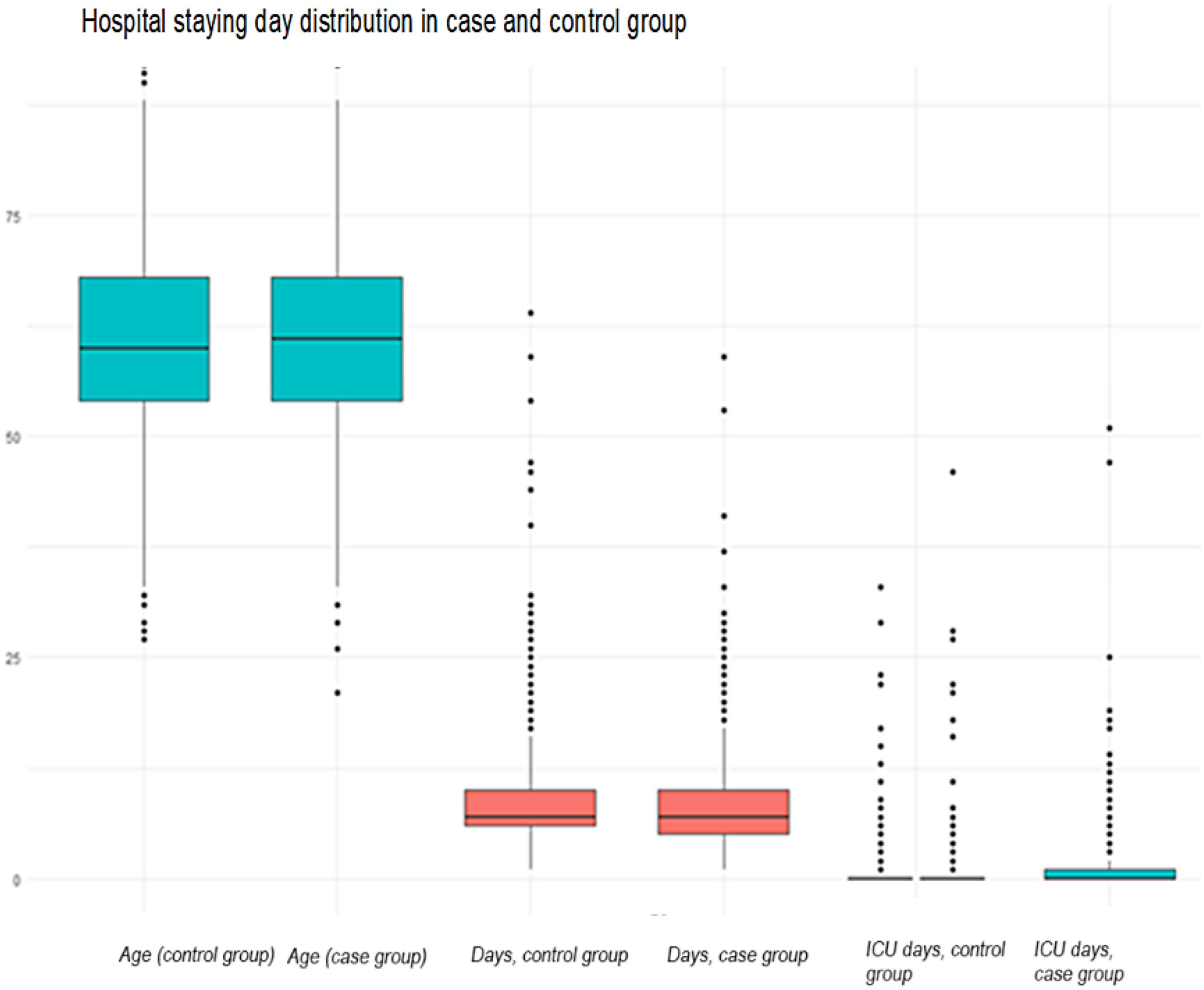
Distribution of hospital length of stay in CPU (case) and non-CPU (control) groups.

The median hospital length of stay was 7.00 [6.00; 10.00] days in the non-CPU group and 7.00 [5.00; 10.00] days in the CPU group. Although the difference reached statistical significance (*p* = 0.001), the absolute difference between groups was minimal (within approximately one day) and was not considered clinically meaningful (Figure 3).

### Direct costs

Based on available data, a comparative analysis of direct treatment costs between the study groups was performed. Reimbursement per case remained unchanged across the study periods. A statistically significant difference in costs was observed between the groups (t = −5.65, df = 3,016.4, *p* < 0.001).

The median direct cost per case was 216,206.26 [216,206.26; 893,312.59] tenge in the non-CPU group and 893,312.59 [240,918.07; 995,415.89] tenge in the CPU group.

### Mortality rate

**Figure 4:**
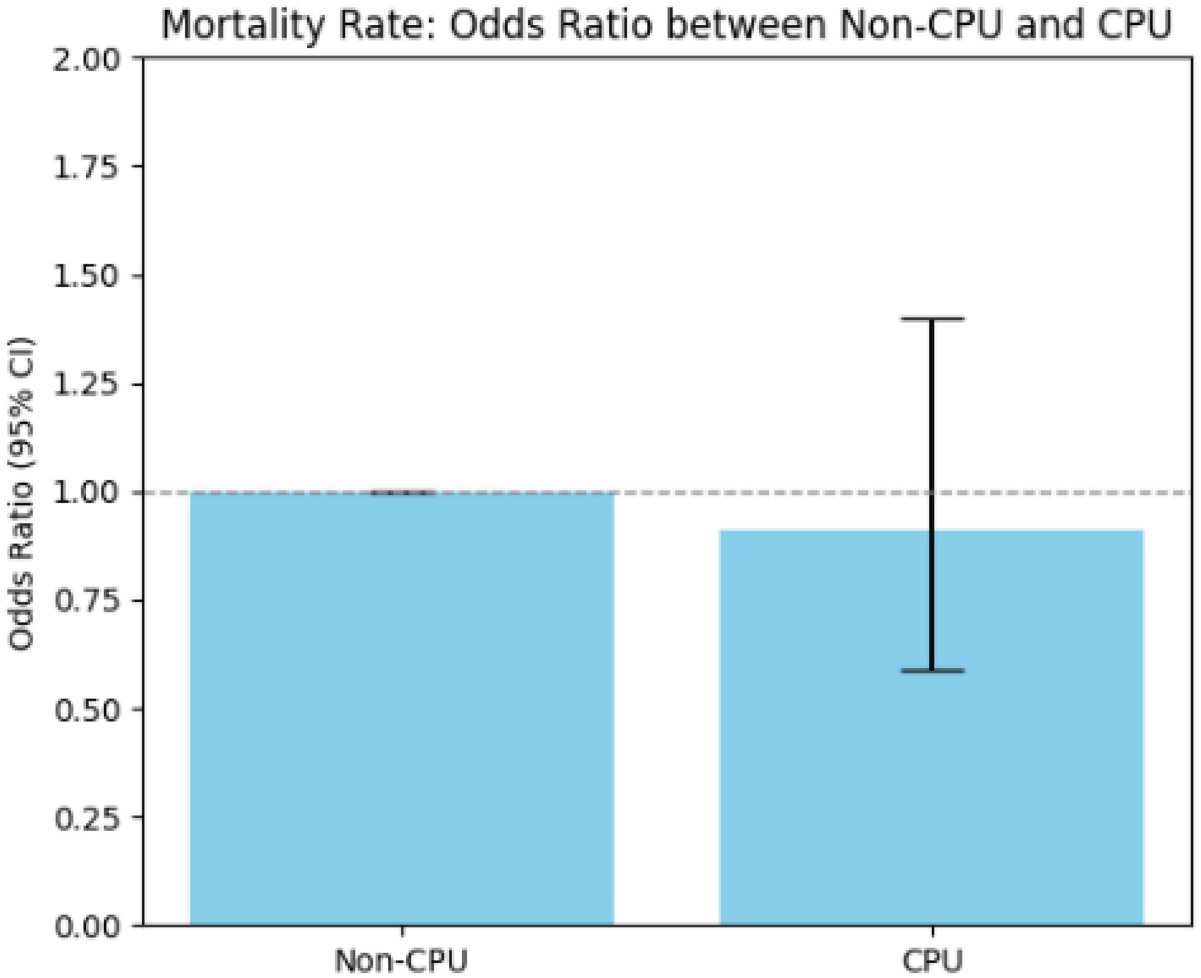
Mortality rates in control and case group.

A comparative analysis of crude in-hospital mortality was performed between the study groups. No statistically significant difference in mortality was observed between the non-CPU and CPU groups [38 [2.4%] vs. 46 [2.2%]; *p* = 0.543], corresponding to an odds ratio of 0.91 (95% CI: 0.59–1.40). Figure 4 illustrates the odds ratios for mortality between the comparison groups.

### Adjusted analysis

To account for baseline differences between the study groups, a propensity score–adjusted logistic regression analysis was performed. The propensity score was estimated using age, sex, diagnosis, and procedural interventions as covariates.

After adjustment for these variables and the propensity score, implementation of the Chest Pain Unit was associated with improved in-hospital survival (adjusted OR = 1.556).

## Discussion

Our study demonstrated that implementation of a CPU was associated with changes in diagnostic and management patterns among patients presenting with acute chest pain. Patients managed in the CPU were more likely to receive a diagnosis of acute myocardial infarction, which may reflect differences in diagnostic pathways, cardiology-oriented monitoring, and patient selection.

A higher proportion of men was observed in our cohort, which is consistent with previous studies showing that ACS is more common in male patients, although women often present at older age and with greater comorbidity burden (18–21).

The observed differences in revascularization patterns between the CPU and non-CPU groups may suggest that the introduction of a dedicated chest pain pathway may facilitate more targeted use of invasive strategies and more timely treatment of patients with clear indications for intervention. Previous studies similarly demonstrated that structured chest pain pathways may influence revascularization decisions and improve clinical workflow, although their impact on long-term outcomes remains uncertain (22–24).

Our findings also support prior evidence showing that specialized chest pain pathways improve operational efficiency. Previous studies have suggested that structured chest pain pathways may improve operational processes, including reductions in unnecessary admissions and shorter hospital stays (12, 25–31). Although a shorter hospital stay was observed in the CPU group in our preliminary study (32), the present analysis was not specifically designed to comprehensively evaluate healthcare utilization or operational efficiency.

Direct in-hospital costs were higher in the CPU group, likely reflecting greater use of diagnostic and interventional procedures. Previous studies have suggested that structured chest pain pathways may be associated with favorable long-term economic outcomes (12, 25, 26). In our study long-term healthcare utilization and cost-effectiveness were not assessed.

The strengths of our study include a large sample size comprising patients managed in both the traditional emergency department and CPU settings. However, several limitations should be acknowledged. First, the retrospective single-center design may limit the generalizability of the findings. Second, despite propensity score–based adjustment, residual confounding cannot be excluded; in particular, differences in baseline diagnosis, including a higher proportion of acute myocardial infarction in the CPU group, may have influenced the rates of revascularization procedures such as PCI. Third, detailed comorbidity data was not available and therefore could not be included in the analysis. Finally, long-term outcomes, including readmission rates, as well as patient- and provider-reported satisfaction, were not assessed.

## Conclusion

Implementation of the Chest Pain Unit model was associated with substantial changes in the evaluation and management of patients presenting with chest pain, including higher rates of acute myocardial infarction identification and PCI utilization compared with the traditional emergency department approach. These findings suggest that CPU-based pathways may improve the organization and intensity of diagnostic assessment in patients with suspected ACS. However, given the observational design of the study, causal inferences regarding clinical effectiveness and economic benefit should be made with caution. Further prospective studies incorporating clinical outcomes and cost-effectiveness analyses are warranted.

## Data Availability

The raw data supporting the conclusions of this article will be made available by the authors, without undue reservation.
